# Activation of trigeminal ganglion satellite glial cells in CFA-induced tooth pulp pain in rats

**DOI:** 10.1371/journal.pone.0207411

**Published:** 2018-11-12

**Authors:** Helena F. Filippini, Paulo A. Scalzilli, Kesiane M. Costa, Raquel D. S. Freitas, Maria M. Campos

**Affiliations:** 1 Programa de Pós-graduação em Odontologia, Escola de Ciências da Saúde, PUCRS, Porto Alegre, RS, Brasil; 2 Laboratório de Patologia, Escola de Ciência da Saúde, PUCRS, Porto Alegre, RS, Brasil; 3 Centro de Pesquisa em Toxicologia e Farmacologia, Escola de Ciências da Saúde, PUCRS, Porto Alegre, RS, Brasil; 4 Programa de Pós-graduação em Medicina e Ciências da Saúde, Escola de Medicina, PUCRS, Porto Alegre, RS, Brasil; Università degli Studi della Campania, ITALY

## Abstract

This study further investigated the mechanisms underlying the rat model of tooth pulp inflammatory pain elicited by complete Freund’s adjuvant (CFA), in comparison to other pulpitis models. Pulps of the left maxillary first molars were accessed. In the CFA group, the pulps were exposed, and CFA application was followed by dental sealing. In the open group, the pulps were left exposed to the oral cavity. For the closed group, the pulps were exposed, and the teeth were immediately sealed. Naïve rats were used as negative controls. Several parameters were evaluated at 1, 2, 3 and 8 days. There was no statistical significant difference among the groups when body weight variation, food or water consumption were compared. Analysis of serum cytokines (IL-1β, TNF or IL-6) or differential blood cell counts did not reveal any evidence of systemic inflammation. The CFA group displayed a significant reduction in the locomotor activity (at 1 and 3 days), associated with an increased activation of satellite glial cells in the ipsilateral trigeminal ganglion (TG; for up to 8 days). Amygdala astrocyte activation was unaffected in any experimental groups. We provide novel evidence indicating that CFA-induced pulp inflammation impaired the locomotor activity, with persistent activation of ipsilateral TG satellite cells surrounding sensory neurons, without any evidence of systemic inflammation or amygdala astrogliosis.

## Introduction

Dental pain associated with tooth pulp inflammation is very common. During the last decades, there were great advances concerning the identification of biomarkers associated with inflammatory pulp pain [1,2]. Classical rodent models of tooth pulp inflammation involve the pulp exposure, with or without the application of phlogistic agents such as formalin, mustard oil, capsaicin, or bacterial lipopolysaccharide (LPS). These models have been essential to elucidate diverse peripheral and central mechanisms underlying dental pain and trigeminal nociceptive transmission [3–6]. However, the knowledge regarding the different models of inflammatory dental pain is still incomplete and further experimental studies on this topic are required.

Complete Freund’s adjuvant (CFA) is a mixture of mineral oils containing heat-inactivated *Mycobacterium tuberculosis*, which has been widely used to induce chronic inflammatory pain when injected into the rat hind paw or knee joint [7,8]. This pro-inflammatory agent has also been used to study orofacial pain, after injection into the temporomandibular joint (TMJ) or perioral skin [9–12]. A few studies have also analyzed the effects of CFA application into the rat tooth pulp. In two of these studies, the authors investigated the mechanisms involved in the ectopic persistent pulp pain or masseter hypersensitivity, by applying CFA into the pulp of the first upper molar, and capsaicin to the pulp of the second molar or into the masseter muscle, respectively [13,14]. Additionally, the application of CFA into the pulp of the right first and second maxillary molars caused an up-regulation of the vesicular glutamate transporter-2 (VGLUT2) in the pulp tissue or trigeminal ganglion (TG) [15]. Noteworthy, the application of CFA to the left lower molar resulted in tongue-referred pain, likely via an up-regulation of Toll-like receptor 4 (TLR4) in neurons of the TG [16].

The orofacial region is predominantly innerved by primary afferent nerve fibers of the trigeminal nerve, and the cell bodies of most trigeminal primary afferents are in the trigeminal ganglion (TG). Interactions between satellite glial cells (SGC) and TG neurons have been shown to play an important role in inducing ectopic discharges in the primary afferents that produce an abnormal sensory input into the central nervous system that can result in a chronic pain state following trigeminal nerve injury or inflammation [17–22]. SGC envelop the primary afferent neurons in sensory ganglia, provide metabolic and structural support of the neurons and can modulate their excitability [23]. The neuron-glia interaction is a key mechanism in orofacial pain [20,21]. The glial interleukin-1β in SGC plays an important role in ectopic tooth pain in adjacent tooth [24] and upregulates neuronal sodium channel 1.7 in trigeminal ganglion [25].

Amygdala is a complex brain structure. As part of the limbic system, it is involved in emotional responses, affective states and pain processing [26,27]. The role of amygdala in neuropathic pain is widely studied [28–31], although the role of this anatomical structure in tooth pain is still unclear. In orofacial region, an interesting evidence showed that tooth loss leads to increased gray matter volume in several cognitive and limbic brain zones and the amygdala was one of these regions [32]. In contrast, a human research found a decreased of gray matter volume in the bilateral amygdala of subjects with trigeminal neuralgia [33]. Using optogenetics and brain slice physiology, a recent study analyzed the cortico-amygdala transmission in an arthritis pain model in male rats. The different neurons from amygdala were examined and the results showed that the infralimbic amygdala pathway controls the activity of medial prefrontal cortex [31].

Glial cells in the central nervous system (CNS) also participate in the development and maintenance of inflammatory and neuropathic pain. Studies suggested that microglia and astrocytes are involved in synaptic function modulation, as well as, neuronal excitability [23, 34,35,36]. A recent study showed the participation of amygdala microglial cells in pain processing. The authors showed that inhibition of microglia in the basolateral amygdala enhanced the analgesic effects of morphine, probably due to the activation of the GABAergic system [37]. On the other hand, the role of astrocytes in this structure in dental pain is still unclear.

The present study aimed to further characterize the mechanisms underlying CFA-induced tooth pulp inflammatory pain. The outcomes were evaluated on days 1, 2, 3 and 8 after surgical dental procedures, and were compared to the changes observed in the classical model of inflammation elicited by pulp exposure to the oral cavity.

## Material and methods

### Animals

Male Wistar rats (8-weeks-old, weighing 180 to 200 g) were obtained from the Central Animal House of the Pontifícia Universidade Católica do Rio Grande do Sul (CeMBE; PUCRS; Brazil). The animals were housed under standard conditions of temperature (22 ± 2°C), light (12-h light-dark cycle) and humidity (50–70%), in ventilated cages, with autoclaved wood chip bedding. Standard rodent chow and tap water were provided *ad libitum*, except during the experimental sessions. The surgical dental procedures, the behavioral assessments and the sample collection were carried between 8:00 AM and 5:00 PM. Sample size (N = 4–8 per group, depending on the evaluated parameter) was established based on previous studies [14,16].

The Ethics Committee on Animal Use of Pontifical Catholic University of Rio Grande do Sul (CEUA-PUCRS) evaluated and approved the research project “Activation of trigeminal ganglion satellite glial cells in CFA-induced tooth pulp pain in rats” registered as CEUA 13/00362. The experimental protocols followed the current Brazilian guidelines for the care and use of animals for scientific and didactic procedures, from the National Council for the Control of Animal Experimentation (CONCEA, Brazil). We followed the ARRIVE Guidelines to report *in vivo* experiments [38]. The number of animals and the intensity of noxious stimuli were the minimum necessary to demonstrate the consistent effects.

### General protocols and distribution of groups

**Fig 1:**
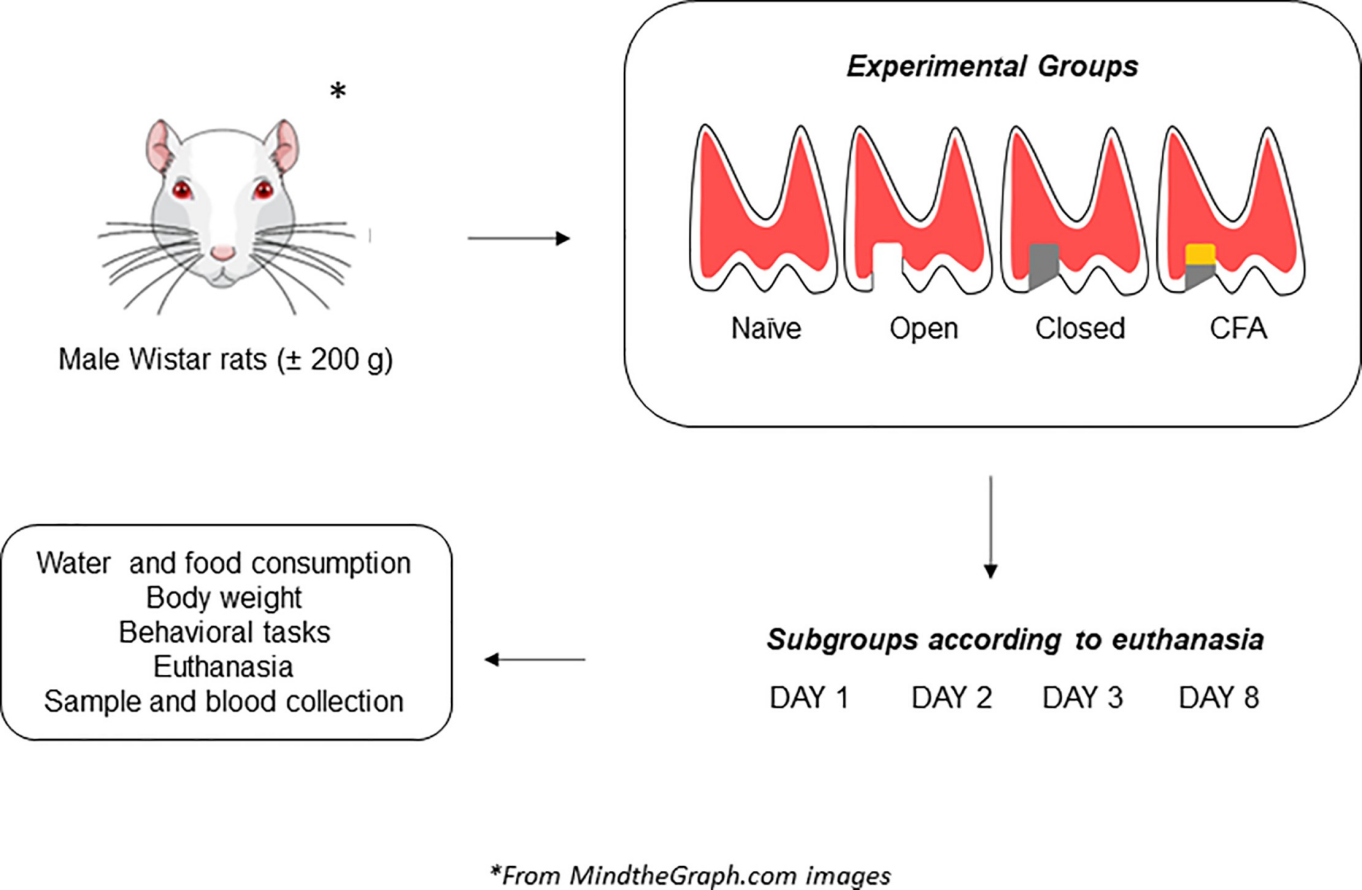
Representation of the general protocols used in the present study. The rats were assigned into four experimental groups, namely naïve, open, closed or CFA. Distinct subgroups were euthanized at 1, 2, 3, or 8 days after the tooth pulp intervention. Several parameters were evaluated in each experimental time, and the appropriate samples were collected.

The induction of tooth pulp inflammatory pain was adapted from the method described by Shimizu et al. [14]. The animals were anesthetized by an i.p. injection of xylazine (10 mg/kg) plus ketamine (100 mg/kg). There were 16 groups. The rats were initially assigned into four experimental groups. *Naïve*, open, closed and CFA. Then, these groups were divided in additional 4 subgroups, depending on the euthanasia time (1, 2, 3, and 8 days). The group named *naïve* had no dental procedure. For the other experimental groups, the pulps of the left maxillary first molars were surgically exposed with a 1/2 size drill in low-speed rotation, under irrigation. In the group named *open*, the pulps were left exposed to the oral cavity, as this is a reference model to study pulp inflammatory pain [39]. In the group named *closed*, the pulps were exposed, and the teeth were immediately sealed with temporary dental filling [40]; this group was included to assess the effects of dental closing, without CFA application. In the last group, denoted *CFA*, the pulps were exposed, and CFA (Sigma, MO, USA; 1 mg/ml; heat-killed and dried *M*. *tuberculosis*, each milliliter of vehicle containing 0.85 ml paraffin oil plus 0.15 ml mannide monooleate; 1:1 oil/saline emulsion) was applied into the pulp, with a soaked small piece of paper-point for 1 min, followed by dental sealing. On days 1, 2, 3, and 8 after dental procedures, body weight variation (in g), food and water consumption/day/animal (in ml and g, respectively) were registered. Subsequently, the animals were evaluated in the open-field test, as described below. Following the behavioral sessions, the animals were euthanized by inhalation of sevoflurane, and a blood sample was collected for hematological differential cell counts. The left maxillae were removed for hematoxylin and eosin (H&E) staining. Serum, left and right TG and amygdala were also stored for further analysis of cytokine levels and GFAP-immunopositivity, as described below. A scheme showing the general protocols used in the present study is depicted in the Fig 1. In this study, six animals were lost during the tooth opening procedure, whereas two animals were euthanized according to the humane endpoints (postoperative distress).

### Behavioral assessment in the open-field

Alterations indicative of locomotor (crossing), exploratory (rearing) and pain-like behavior (facial grooming) were registered in the open-field arena [41]. Different subgroups of animals from all experimental groups (naïve, open, closed and CFA) were evaluated at 1, 2, 3, or 8 days after dental procedures. They were individually placed in the center of a plywood box (34.5 cm height x 249 cm circumference x 77.5 cm radius), with the floor divided into 17 squares. Duration of grooming (s) was cumulatively registered over 5 min. The number of rearing movements and the number of squares crossed with the four paws were also registered.

### Determination of cytokine levels

The serum levels of pro-inflammatory cytokines IL-1β, TNF and IL-6 were measured in order to further assessing the possible systemic inflammatory changes following the induction of tooth pulp inflammation by CFA. For this purpose, the blood samples were collected by cardiac punction. The samples were centrifuged at 1300 x g at 4°C for 10 min. The supernatant was rapidly frozen and stored at -80°C for analysis of the pro-inflammatory cytokines interleukin-1β (IL-1β), tumor necrosis factor (TNF), and interleukin-6 (IL-6), using specific enzyme-linked immunosorbent assay (ELISA) kits, according to the recommendations of the supplier (R&D Systems; Minneapolis, Minnesota, USA).

### Hematologic parameters

For determination of differential white blood cells, the samples were collected by cardiac punction [41]. Immediately after, a blood drop was taken for smear evaluation, by May-Grunewald-Giemsa staining. Differential counts (neutrophils, eosinophils, basophils, lymphocytes, monocytes, and immature cells) were estimated under a ×40 objective (Olympus CH30 model), by counting 100 cells.

### Histological analysis

To confirm the induction of tooth pulp inflammation, the left maxillae were collected and fixed in 10% neutral-buffered formalin solution. The samples were decalcified with 5% nitric acid (pH 7.4). The paraffin blocks containing the maxillae were serially cut (4 μm-thickness) in the longitudinal plane. The sections were stained with H&E, and qualitatively examined under light microscopy. A microscope (Axio Imager A1) coupled to an image capture system (Axio Vision Rel. 4.4 Software Multimedia), from Carl Zeiss (Hallbergmoos, Germany) was used (x200 magnification).

#### Evaluation of GFAP-immunopositivity in TG and amygdala

The TG and the amygdala (ipsilateral and contralateral) were removed for immunohistochemistry analysis to determine GFAP-positive cells. Immunohistochemistry was performed on paraffin tissue sections (4-μm thickness) by using the monoclonal rabbit anti-GFAP (1:250, Cat. #04–1062; Lot #2145973; Merck Millipore, Germany), according to the method described previously [42–44]. High-temperature antigen retrieval was performed by immersion of the slides in a water bath at 98–100°C in 10 mM trisodium citrate buffer, Tris-EDTA buffer pH 9.0 (anti-GFAP) for 40 min. The peroxidase was blocked by incubating the sections with perhidrol 5% for 30 min. The nonspecific protein binding was blocked with milk serum solution 5% for 30 min. After overnight incubation at 4°C with the primary antibody anti-GFAP, the slides were washed with PBS and incubated with the secondary antibody HRP conjugate (Invitrogen, CA, USA), ready-to-use, for 20 min at room temperature. The sections were washed in PBS, and the visualization was completed by using 3,3′-diaminobenzidine (Dako Cytomation, CA, USA) in chromogenic solution and counterstained lightly with Harris’s Hematoxylin solution. Images were examined with a Zeiss AxioImager M2 light microscope (Carl Zeiss, Germany). The images were captured in x200 magnification. The number of GFAP-positive astrocytes (amygdala) and the number of neurons surrounded by GFAP-immunopositive satellite cells (TG) were counted by two different independent examiners, in a blinded manner.

### Statistical analysis

Data regarding the body weight, water and food consumption are expressed as the mean ± the standard error mean. The behavioral and immunohistochemistry data are expressed as the median accompanied by the upper and lower quartiles and the minimum and the maximum values. For statistical analysis of data, the non-parametric Kruskal-Wallis test, followed by Dunn's multiple comparison test was used. P values less than 0.05 were considered statistically significant.

## Results

### General analysis

**Fig 2:**
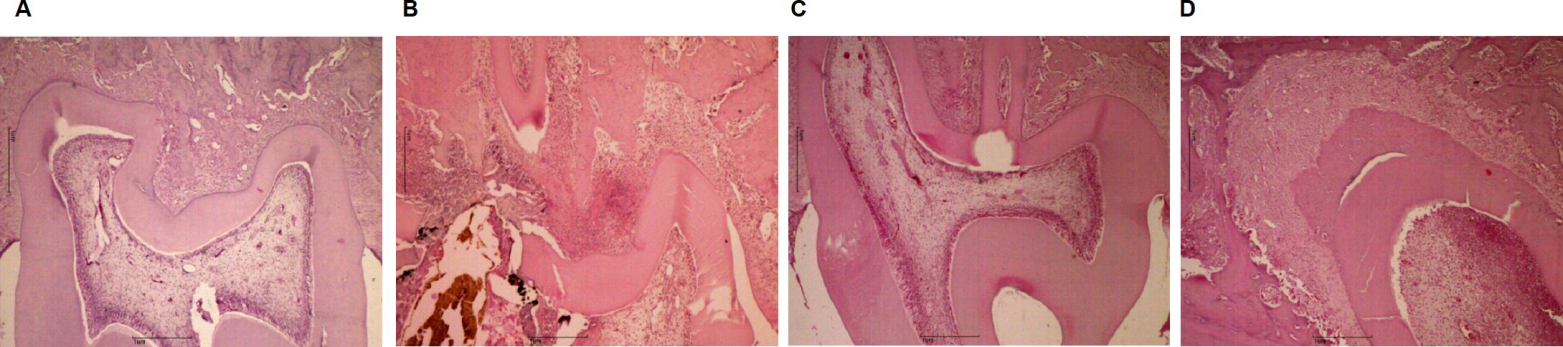
H&E-stained sections of left first molars. Naïve (A), open (B), closed (C) or CFA (D) groups, at three days after the surgical dental procedures.

A qualitative analysis of H&E-stained sections (Fig 2A–2D) showed the presence of inflammatory cells in the pulp tissue of all the experimental groups, in comparison to the naïve group (Fig 2A), according to the evaluation at three days after dental procedure. The CFA group displayed a higher pulp inflammatory infiltrate, in relation to the open and closed groups (Fig 2B and 2C).

**Fig 3:**
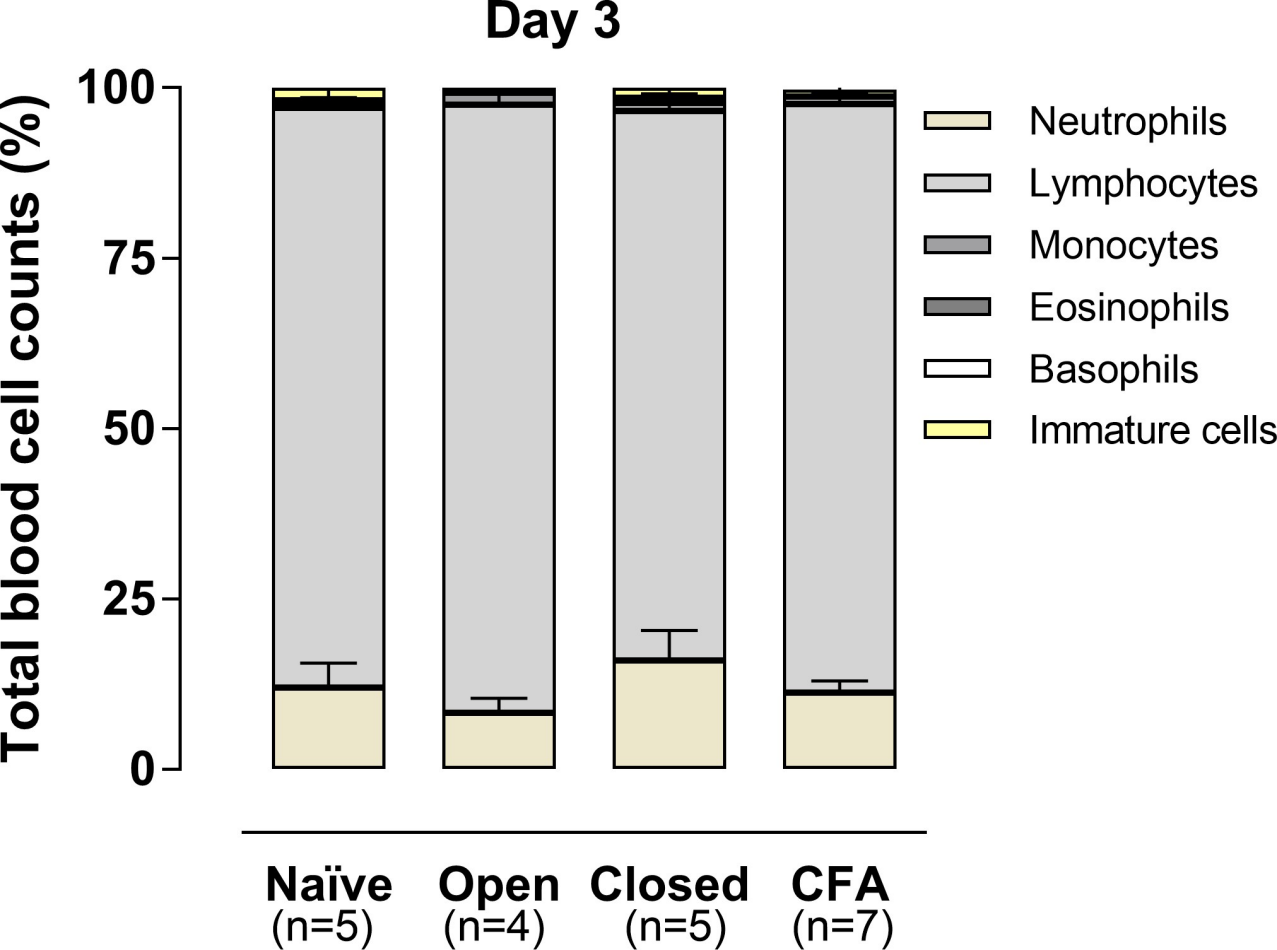
Total blood cell counts evaluated on the third day. The columns represent the mean of 5–7 experiments, and the vertical lines indicate the standard error of mean (P>0.5; Kruskal-Wallis plus Dunn’s test).

The analysis of the peripheral blood cells showed a predominance of lymphocytes in all the groups with pulp inflammation, without great variations of the neutrophil numbers. A similar profile was observed in naïve rats (Fig 3).

**Fig 4:**
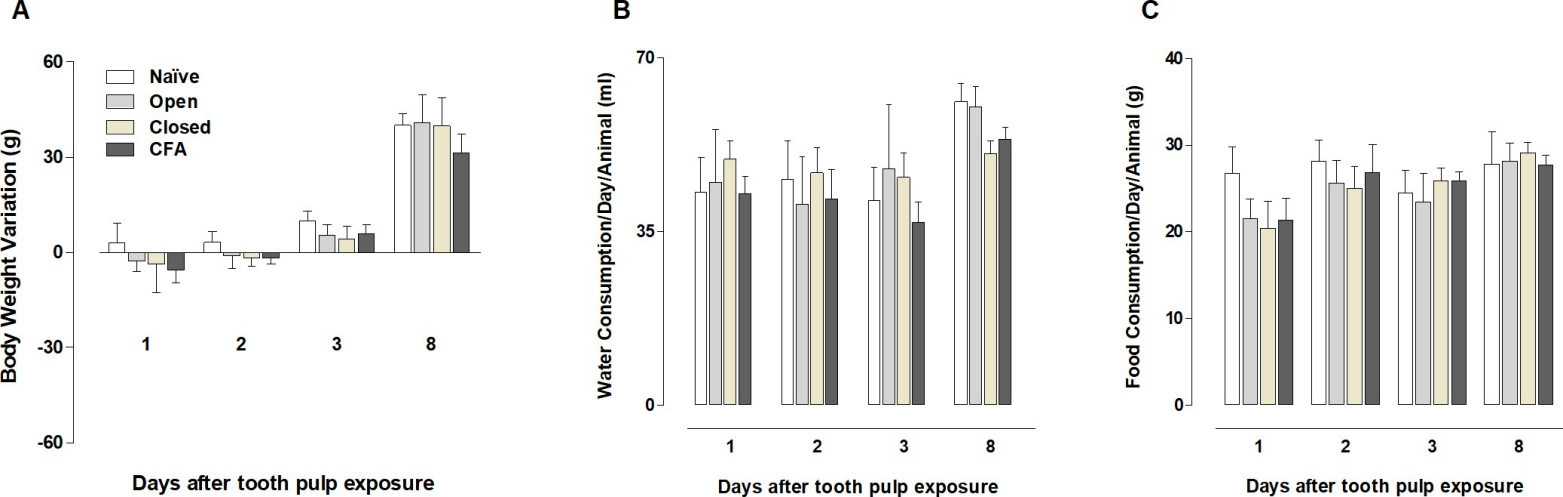
**Body weight variation (A), water (B) and food (C) consumption per animal/day in the different experimental groups.** The columns represent the mean of 5–7 experiments, and the vertical lines indicate the standard error of mean. (P> .05; Kruskal-Wallis plus Dunn’s test).

The body weight gain was slightly reduced in animals of open, closed and CFA groups from days 1 to 3 after pulp access (Fig 4A), although the differences were not statistically significant when compared to the naïve controls (*P* > .05). The water consumption/animal trended toward a decrease in animals from the closed and CFA groups on the 8^th^ day (*P* > .05; Fig 4B). No significant difference was detected when comparing the food consumption/day/animal in any experimental time, although an apparent reduction can be observed at day 1, in all the groups with tooth pulp access (*P* > .05; Fig 4C). The serum levels of the inflammatory cytokines (IL-1β, TNF and IL-6) were not detectable in any of the analyzed groups (S1 Table).

### Behavioral changes in the open-field test

**Fig 5:**
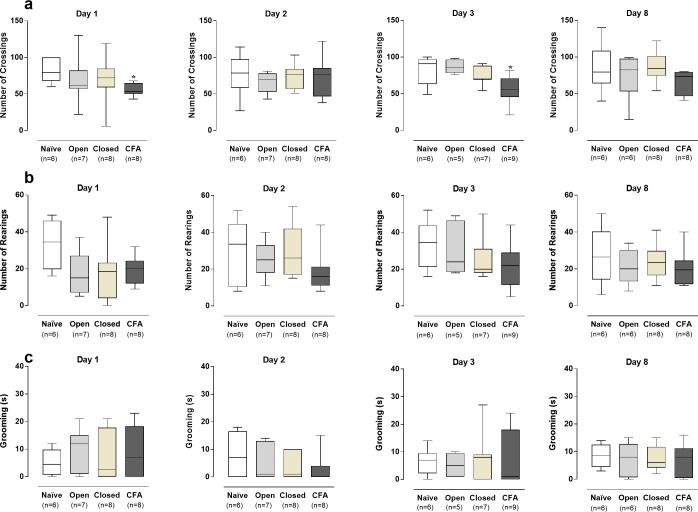
Behavioral assessment in the open-field arena. Crossing (A) and rearing (B) counts, and facial grooming time in seconds (C) of rats distributed into the different experimental groups, at 1, 2, 3, or 8 days after the dental surgical procedures. The box plots show the median of 5–8 experiments with the upper and lower quartiles. The whiskers indicate the maximal and the minimal values. (*P < .05; Kruskal-Wallis plus Dunn’s test).

The locomotor activity, as indicated by the number of crossings in the open-field arena, was significantly reduced (*P* < .05) in the CFA-inflamed group, but not in the open or closed groups, on days 1 and 3 after the application to the dental pulp (Fig 5A). No significant variations were seen on days 2 and 8 of evaluation. The number of rearings and the facial grooming time were not significantly different when comparing all the experimental groups to the naïve controls (Fig 5B and 5C; *P* > .05).

### TG satellite glial cells activation

**Fig 6:**
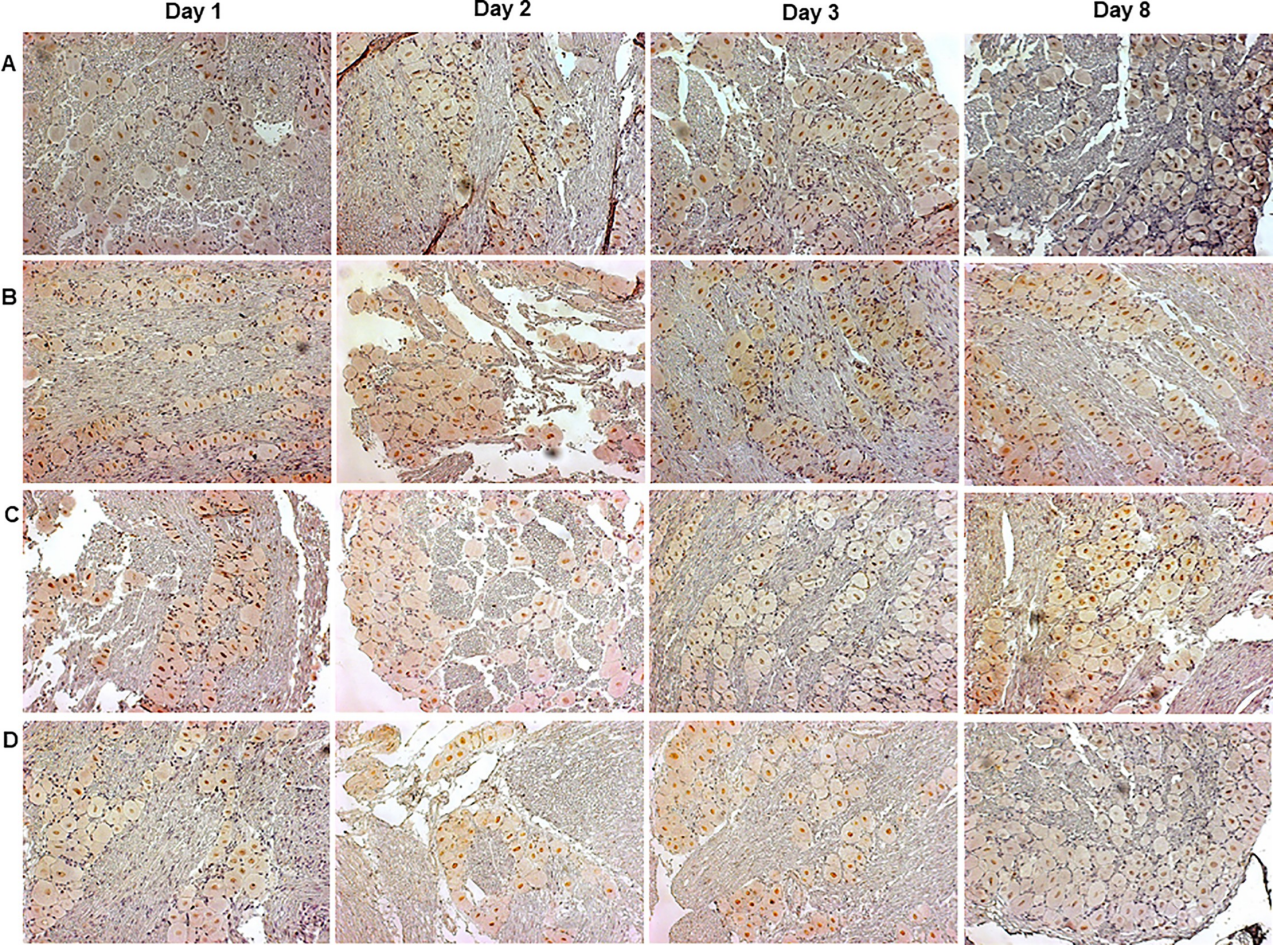
Neurons encircled by GFAP-immunopositive satellite cells, in the left trigeminal ganglion of rats. Naïve (A), open (B), closed (C) and CFA (D) groups, at 1, 2, 3 and 8 days after procedures.

The application of CFA to the pulp of the left first molars led to an increased activation of satellite cells in the ipsilateral TG, from days 1 to 8 after the surgical dental procedures, as indicated by the density of neurons enclosed by GFAP-positive satellite cells, in the representative images (Fig 6).

**Fig 7:**
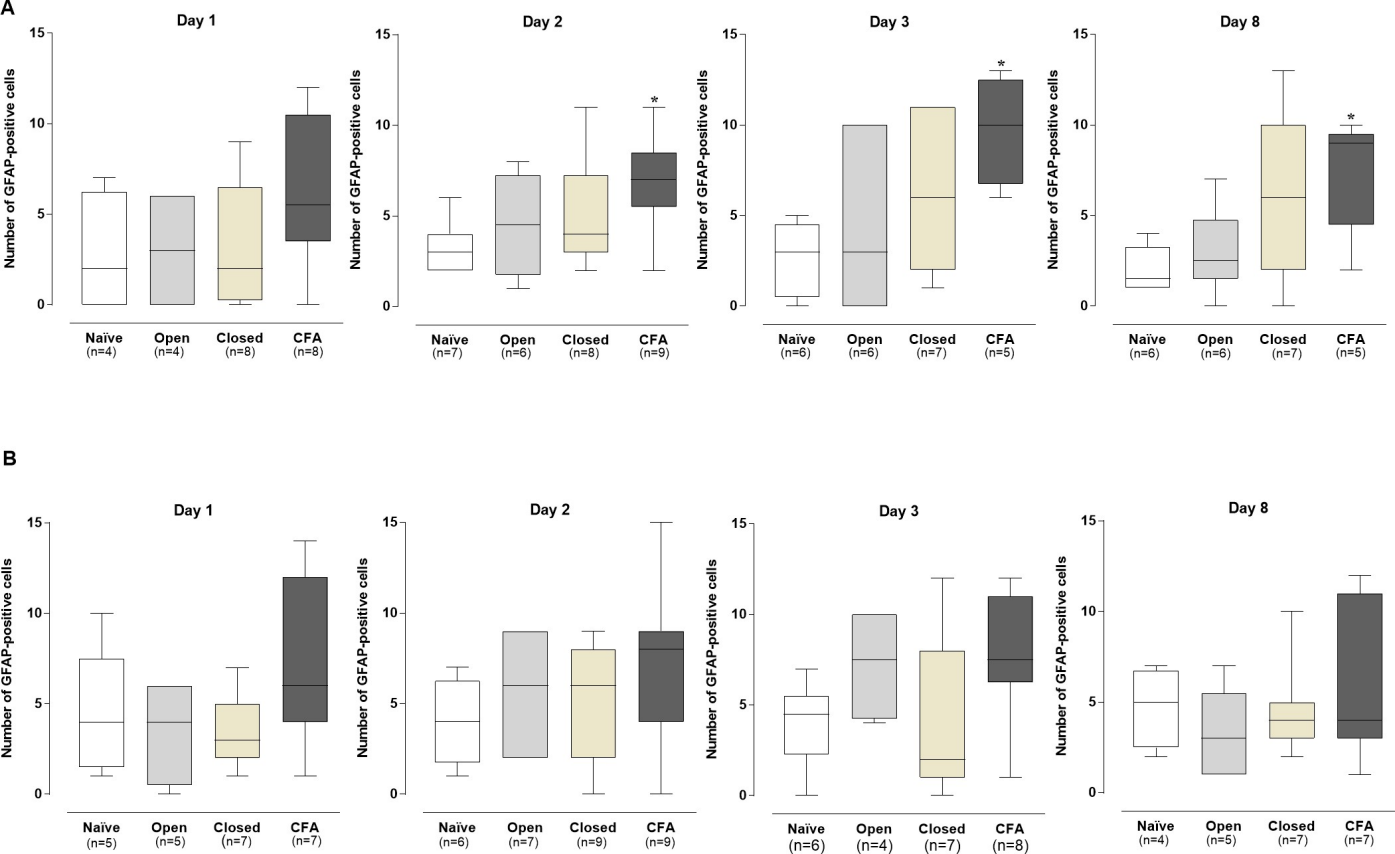
**GFAP-immunopositive glial satellite cells in the ipsilateral (A) and contralateral (B) trigeminal ganglion in the different experimental groups.** The box plots show the median of 4–7 experiments with the upper and lower quartiles. The whiskers indicate the maximal and the minimal values. *P < .05 (Kruskal-Wallis plus Dunn’s test).

The differences in comparison to the naïve group were statistically significant on days 2, 3, and 8 (*P* < .05; Fig 7A), according to the quantitative analysis. Conversely, the number of neurons with activated satellite cells was not altered in the contralateral TG obtained from CFA-treated animals, when compared to naïve rats (Fig 7B). No significant differences in the numbers of neurons with GFAP-positive satellite cells were seen in the open or closed groups, according to the evaluation of the ipsi- or contralateral TGs (*P* > .05; Fig 7A and 7B)

### Evaluation of astrocyte activation in amygdala

**Fig 8:**
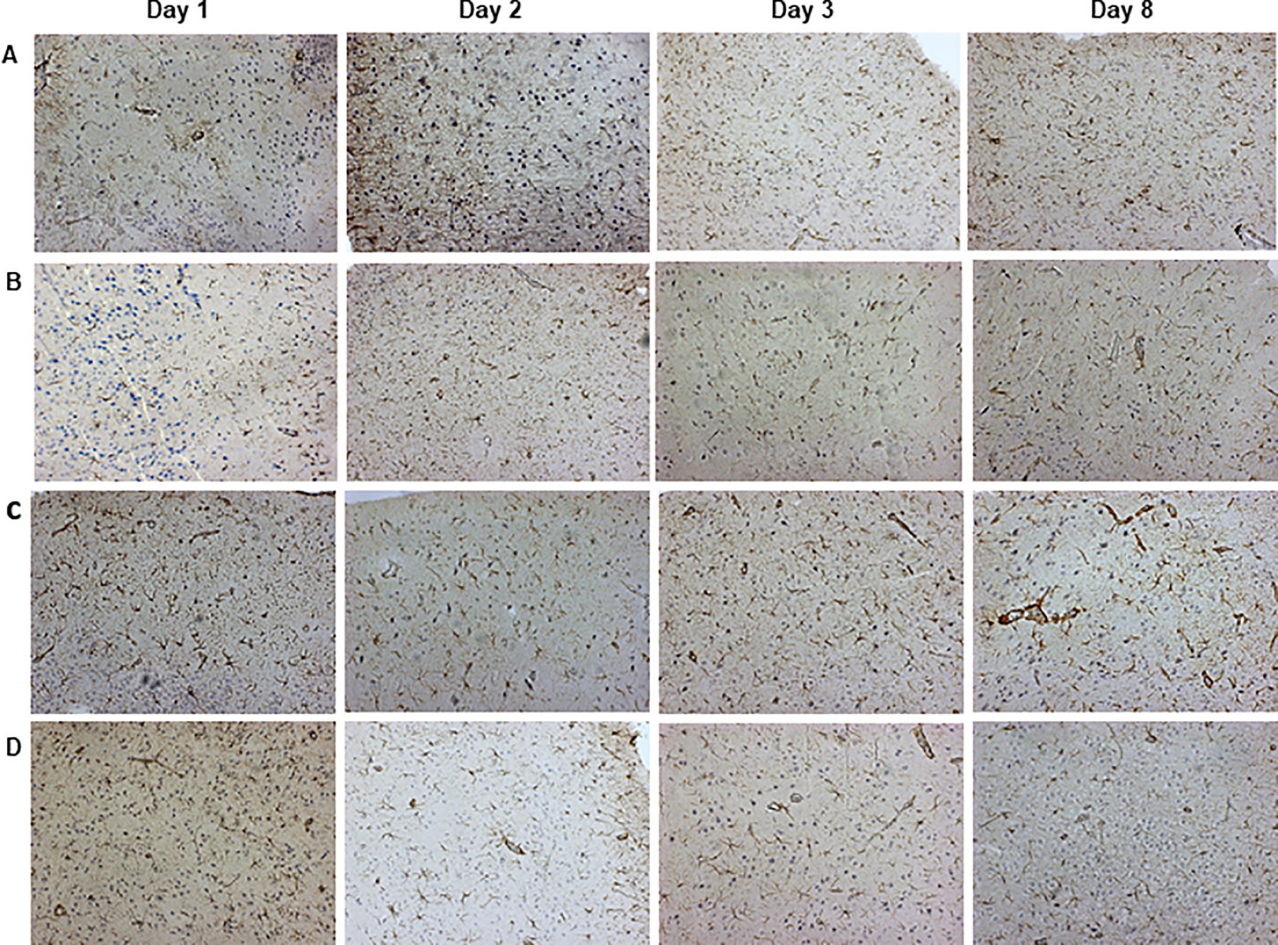
Representative images of immunohistochemical analysis for GFAP-positive astrocytes in the left amygdala of rats. Naïve (A), open (B), closed (C) and CFA (D) groups, at 1, 2, 3 and 8 days after procedures.

The amygdala has been described as an important site for processing of the emotional components of pain [29]. Here, we assessed the astrocyte activation, by determining the number of GFAP-immunopositive cells, in the left and right amygdalas of animals subjected to different models of tooth pulp inflammation. Similar levels GFAP-immunopositive astrocytes were observed in the representative images of the left amygdala, for all the experimental groups, at different time-points (Fig 8).

**Fig 9:**
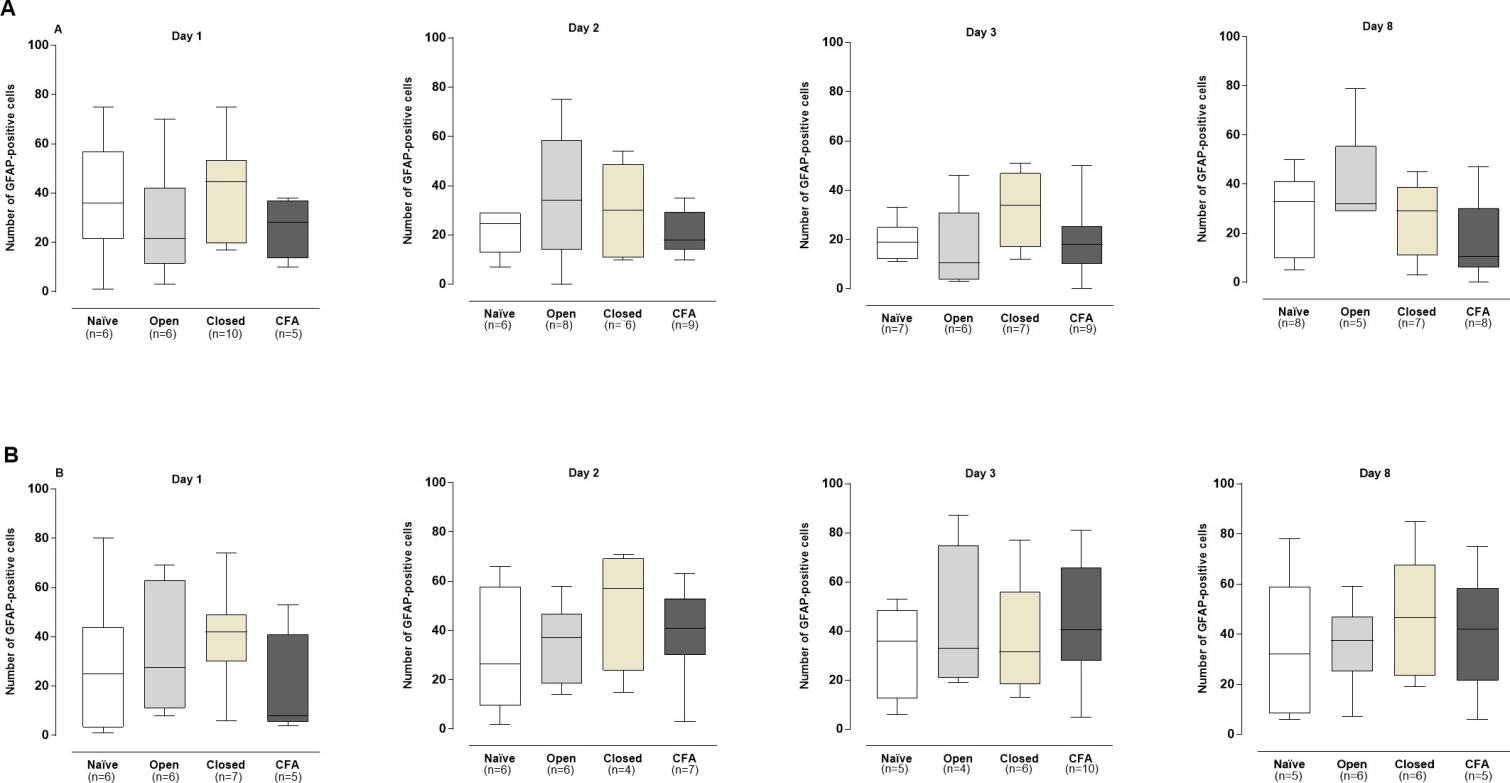
**GFAP-immunopositive astrocytes in the ipsilateral (A) and contralateral (B) amygdala in the different experimental groups**. The box plots show the median of 4–7 experiments with the upper and lower quartiles. The whiskers indicate the maximal and the minimal values (P>.05; Kruskal-Wallis plus Dunn’s test).

The quantitative analysis did not reveal any detectable difference in the numbers of activated astrocytes in the ipsi- or contralateral amygdalas of all the experimental groups, in relation to the naïve controls (P > .05; Fig 9A and 9B).

## Discussion

Classical rodent models of inflammatory dental pain involve the pulp exposure, with or without the application of inflammatory agents. These models helped to elucidate the mechanisms underlying dental pain and trigeminal nociceptive transmission [3–6]. However, the knowledge regarding the involvement of glial cells and amygdala in different models of inflammatory dental pain is still incomplete. Therefore, this study assessed the involvement of SGC and astrocytes in TG and amygdala in the CFA model of inflammatory tooth pain in rats.

The application of CFA into different sites has been widely used to induce inflammation and to characterize the mechanisms implicated in inflammatory pain [8–12]. However, only a few studies investigated the outcomes after the application of CFA into the rat dental pulp [13–16;22]. Our first set of data demonstrated that all surgical groups showed a mild reduction of body weight variation, from the 1^st^ to the 3^rd^ day after the tooth pulp access. Although this data was not statistically different, there are important variations when comparing the experimental groups to the naïve controls. Our results are somewhat contradictory to that showed by Shimizu et al. (2014), describing no alterations of body weight, general behavior, and feeding in rats submitted to CFA tooth pulp inflammation, according to the evaluation at 3 days and 6 weeks [14]. However, the results presented by the authors are descriptive and no graphical data comparing the different groups was presented. In our study, we also observed a slight reduction of food consumption for all the groups with pulp access on day 1, and a decrease of water consumption in the closed and CFA groups, on day 8. Accordingly, it was demonstrated that rats that suffered a bilateral pulp exposure, of the first and second lower molars, showed a reduction in food intake at the first day after procedures, associated with an increased meal duration for up to 8 days, as an indicative of nociception [45]. Corroborating this data, the induction of dental pulp injury in mice led to reduced intake of both water and food, associated to a lessening of body weight for up to 2 days after surgical procedures [46]. Additionally, in a study to verify the effects of cannabinoid receptor 2 (CB2) genic deletion on pulpitis-related pain, wild-type (WT) mice, submitted to unilateral pulp exposure of the first upper and lower molars, presented a reduction in body weight after 2 days following surgery, with a slight reduction of food consumption at 1 day [47]. On the contrary, Gibbs et al. demonstrated a reduction in body weight after 2 days of tooth pulp exposure in mice, without marked changes in water or food consumption, except by a preference for consuming a sucrose-rich solution, a behavior that was altered by the anti-inflammatory drug indomethacin. Herein, it was not possible to observe great differences in the CFA group for the parameters of body weight or water and food consumption, in relation to the open and closed groups. This allows suggesting that tooth pulp access, rather than CFA application, affected these aspects [48].

Next, we investigated to what extent CFA-induced tooth pulp inflammatory pain might alter the serum levels of pro-inflammatory cytokines from days 1 to 8. The results revealed undetectable serum levels of IL-1β, TNF or IL-6 in either experimental groups submitted to tooth pulp exposure, as it was observed in naïve rats. This indicates that pulp exposure to the oral cavity, or to CFA, from day 1 to day 8, was not able to elicit systemic inflammatory changes. This notion was confirmed by data on the differential blood cell counts, which revealed high counts of lymphocytes, associated with low neutrophil numbers in all the analyzed groups at 3 days. This hematological feature is usually observed in the absence of systemic inflammatory changes in rodents [41,44]. In contrast to the present results, a study conducted by Li et al. (2015) showed a time-dependent increase in the serum levels of IL-1β, TNF and IL-18, associated with decreased production of melatonin, after exposure of the pulps of the first and second left molars to the oral cavity [49]. Another study showed an increment of serum IL-1β, on the 1^st^, but not on the 3^rd^ day, in rats that suffered pulp exposure of first and second upper molars [50]. The use of an inflammation model involving two dental elements, instead of one tooth, might explain the discrepant data. Furthermore, the used rat strain was also different. Whether or not the application of CFA to the rat pulp might elicit changes in other biomarkers of systemic inflammation remains to be unraveled.

It was demonstrated that pulp exposure of the mouse left first molar to the oral cavity, resulted in increased frequency and duration of facial grooming on days 1 and 3, followed by decreased exploratory activity in the open-field test on days 7 and 14, suggesting a sequential relationship between dental pain and social anxiety in this experimental model [46]. Alternatively, the unilateral pulp exposure of the first upper and lower molars caused discrete behavioral alterations in WT mice, in comparison to CB2 knockout animals, according to evaluation in the open-field [47]. Gibbs, Urban & Basbaum (2013) also demonstrated a discrete reduction of locomotor activity, after pulp exposure of mouse left and right first maxillary molars [48]. A significant reduction of locomotor and exploratory activity, plus an increase of facial grooming time, was observed in rats with pulp exposure of the first and second upper molars, on the 1^st^, but not on the 3^rd^ day after procedures [50]. In our study, there were no marked differences in the grooming time or the exploratory activity (as measured by the rearing number) in any surgical group, in comparison to the naïve rats. This discrepant data might be related to the number of teeth assessed per animal, and the rodent specie evaluated. In our study, rats were used instead of mice. However, CFA-induced tooth pulp inflammation was associated with a significant reduction of the locomotor activity in the open-field test, as revealed by the reduction of the number of crossings on days 1 and 3.

Of note, a publication by Chudler & Byers showed that decline of body weight variation and exploratory activity, associated with increased freezing time and occasional yawning, were proportional to the number of teeth that suffered pulp exposure (3–4 teeth > 2 teeth) [51]. Thus, the higher intensity of CFA-induced tooth pulp inflammation might explain the reduced locomotor activity in the open-field test, suggestive of nociceptive changes, differently from that observed in the open or closed groups.

It is well recognized that orofacial inflammatory pain transmission relies on the activation of both neuronal and non-neuronal trigeminal cells [52]. The cell bodies of TG neurons are surrounded by SGC, which become activated by inflammatory mediators, contributing to pain facilitation [23]. We demonstrated that CFA-elicited tooth pulp inflammation led to increased activation of SGC in the ipsilateral (but not contralateral) TG, as indicated by the higher number of neurons encircled by GFAP-immunopositive cells, with significant differences at 2, 3, and 8 days after procedures, and a minor increase at 1 day. However, there were no significant differences of GFAP-immunopositivity, in the ipsi- or contralateral TGs, of open or closed groups. Thus, CFA-induced tooth pulp inflammatory pain seems to depend on the persistent activation of ipsilateral TG satellite cells. Our results confirm and extend previous evidence showing that CFA application, to the rat first upper molars, increased the counts of GFAP immunopositive satellite cells, in the ipsilateral TG at 3 and 8 days, contributing to ectopic dental pain after capsaicin application to the pulp of the second molar [11, 22]. The pioneer data published by Stephenson & Byers [40] showed an increment in the counts of neurons surrounded by GFAP-immunopositive cells, on days 3 and 7 following different dental injury protocols in rats, proposing this parameter as a valuable tool for the study of trigeminal-related pain. Besides, the mechanical allodynia and the edema formation, induced by an intra-articular injection of CFA into TMJ, was accompanied by increased numbers of GFAP-positive SGC, in the ipsilateral TG, at 1 and 3 days [53]. Also, there was an increase in the number of neurons enrolled by GFAP-immunopositive satellite cells, after the extraction of right maxillary first and second molars in rats, for up to 10 days [39]. Corroborating our data, Liu et al 2018 demonstrated a time-related increase of GFAP-positive SGC surrounding the TG, peaking at 28 days after tooth pulp exposure [54].

As discussed beforehand, the amygdala is an anatomical region linked to the emotional and affective components of pain [27]. It was demonstrated that chronic pain caused by the injection of CFA into the mouse paw, or by the sciatic nerve ligation, was related to the development of anxiety, via modulation of opioid receptors in the amygdala [33]. A more recent study showed that spinal ligation-induced neuropathic pain, in depressed rats submitted to olfactory bulbectomy, led to marked changes in cytokine levels in the amygdala, accompanied by microglia and astrocyte activation [55]. In our study, the induction of tooth pulp inflammation by CFA failed to induce astrocyte activation, according to the evaluation of ipsi- and contralateral amygdala. As well, no astrocyte amygdala activation was observed in the open or closed groups. Noteworthy, a meta-analysis that included studies with functional magnetic resonance imaging, succeeding human pulpal electrical stimulation, revealed the activation of dorsolateral prefrontal cortex, a region implicated in the cognitive-affective network, without any stimulation of amygdala or hippocampus [56]. Maybe, the time used by us to evaluate pain was not enough to involve astrocytes activation in amygdala. Piao et al. (2009) showed that the activation of astrocytes appeared to be delayed compared to microglia in medullary dorsal horn, in a trigeminal neuropathic pain model in rats [35]. The induction of tooth pulp inflammation by CFA application, or even in the open and closed groups, was not correlated with astrocyte activation in the ipsi- or contralateral amygdala, according to the similar numbers of GFAP-positive astrocytes, in relation to the naïve controls. Therefore, tooth pulp inflammatory pain, induced by CFA application or by pulp exposure to the oral cavity, is not likely dependent on amygdala astrogliosis in this experiment model. Additional mechanisms involving the amygdala, and other anatomical regions implicated in the affective-emotional circuitry of pain, remain to be analyzed in the different models of pulpitis.

## Conclusion

In the present study, locomotor decrease, a pain-like behavior, occurred only in rats with CFA-treated pulps and was accompanied by satellite glial cell activation in the trigeminal ganglion. These findings suggest that satellite glial cells in the TG have important role in inflammatory dental pain conditions and may offer new targets for management of these conditions.

## Supporting information

S1 Table Serum levels of pro-inflammatory cytokines in the different experimental groups. (DOC)

## References

[1] Hargreaves KM. Orofacial pain. Pain. 2011; 152: S25–S32. 10.1016/j.pain.2010.12.024 21292394 PMC3077822

[2] Rechenberg DK, Galicia JC, Peters OA. Biological Markers for Pulpal Inflammation: A Systematic Review. PLoS One. 2016; 11: e0167289 10.1371/journal.pone.0167289 27898727 PMC5127562

[3] Byers MR, Närhi MV. Dental injury models: experimental tools for understanding neuroinflammatory interactions and polymodal nociceptor functions. Crit Rev Oral Biol Med. 1999; 10: 4–39. 10759425 10.1177/10454411990100010101

[4] Chidiac JJ, Rifai K, Hawwa NN, Massaad CA, Jurjus AR, Jabbur SJ et al Nociceptive behaviour induced by dental application of irritants to rat incisors: a new model for tooth inflammatory pain. Eur J Pain. 2002; 6: 55–67. 10.1053/eujp.2001.0305 11888229

[5] Sessle BJ. Peripheral and central mechanisms of orofacial pain and their clinical correlates. Minerva Anestesiol. 2005; 71: 117–36. 15756153

[6] Khan A, Hargreaves KM. Animal models of orofacial pain. Methods Mol Biol. 2010; 617: 93–104. 10.1007/978-1-60327-323-7_8 20336416

[7] Holmdahl R, Lorentzen JC, Lu S, Olofsson P, Wester L, Holmberg et al Arthritis induced in rats with nonimmunogenic adjuvants as models for rheumatoid arthritis. Immunol Rev. 2001; 184: 184–202. 12086312 10.1034/j.1600-065x.2001.1840117.x

[8] Koch DA, Silva RB, de Souza AH, Leite CE, Nicoletti NF, Campos MM et al Efficacy and gastrointestinal tolerability of ML3403, a selective inhibitor of p38 MAP kinase and CBS-3595, a dual inhibitor of p38 MAP kinase and phosphodiesterase 4 in CFA-induced arthritis in rats. Rheumatology. 2014; 53: 425–32. 10.1093/rheumatology/ket369 24241037

[9] Imbe H, Iwata K, Zhou QQ, Zou S, Dubner R, Ren K. Orofacial deep and cutaneous tissue inflammation and trigeminal neuronal activation. Implications for persistent temporomandibular pain. Cells Tissues Organs. 200; 169: 238–47. 10.1159/000047887 11455119

[10] Do Nascimento GC, Leite-Panissi CR. Time-dependent analysis of nociception and anxiety-like behavior in rats submitted to persistent inflammation of the temporomandibular joint. Physiol Behav. 2014; 125: 1–7. 10.1016/j.physbeh.2013.11.009 24291383

[11] Kurose M, Imbe H, Nakatani Y, Hasegawa M, Fujii N, Takagi R, et al Bilateral increases in ERK activation at the spinomedullary junction region by acute masseter muscle injury during temporomandibular joint inflammation in the rats. Exp Brain Res. 2017; 235(3): 913–92. 10.1007/s00221-016-4852-9 27933357

[12] Santos BM, Garattini EG, Branco LGS, Leite-Panissi CRA, Nascimento GC. The therapeutic potential of cystathionine gamma-lyase in temporomandibular inflammation-induced orofacial hypernociception. Physiol Behav. 2018 5 1; 188: 128–133. 10.1016/j.physbeh.2018.02.007 29425970

[13] Matsuura S, Shimizu K, Shinoda M, Ohara K, Ogiso B, Honda K, et al (2013). Mechanisms underlying ectopic persistent tooth-pulp pain following pulpal inflammation. PLoS One. 2013; 8: e52840 10.1371/journal.pone.0052840 23341909 PMC3547043

[14] Shimizu K, Matsumoto K, Noma N, Matsuura S, Ohara K, Komiya H. et al Involvement of trigeminal transition zone and laminated subnucleus caudalis in masseter muscle hypersensitivity associated with tooth inflammation. PLoS One. 2014; 9: e109168 10.1371/journal.pone.0109168 25279551 PMC4184877

[15] Yang ES, Jin MU, Hong JH, Kim YS, Choi SY, Kim TH, et al Expression of vesicular glutamate transporters VGLUT1 and VGLUT2 in the rat dental pulp and trigeminal ganglion following inflammation. PLoS One. 2014; 9: e109723 10.1371/journal.pone.0109723 25290694 PMC4188624

[16] Ohara K, Shimizu K, Matsuura S, Ogiso B, Omagari D, Asano M, et al Toll-like receptor 4 signaling in trigeminal ganglion neurons contributes tongue-referred pain associated with tooth pulp inflammation. J Neuroinflammation. 2013; 10: 139 10.1186/1742-2094-10-139 24267924 PMC4222866

[17] Zhang X, Chen Y, Wang C, Huang LY. Neuronal somatic ATP release triggers neuron-satellite glial cell communication in dorsal root ganglia. Proc Natl Acad Sci U S A. 2007;104(23): 9864–9. 10.1073/pnas.0611048104 17525149 PMC1887586

[18] Xie YF, Zhang S, Chiang CY, Hu JW, Dostrovsky JO, Sessle BJ. Involvement of glia in central sensitization in trigeminal subnucleus caudalis (medullary dorsal horn). Brain Behav Immun. 2007; 21(5): 634–41. 10.1016/j.bbi.2006.07.008 17055698

[19] Xie W, Strong JA, Zhang JM. Early blockade of injured primary sensory afferents reduces glial cell activation in two rat neuropathic pain models. Neuroscience. 2009; 160(4): 847–57. 10.1016/j.neuroscience.2009.03.016 19303429 PMC2777638

[20] Iwata K, Katagiri A, Shinoda M. Neuron-glia interaction is a key mechanism underlying persistent orofacial pain. J Oral Sci. 2017; 59(2):173–175. 10.2334/josnusd.16-0858 28637974

[21] Hossain MZ, Unno S, Ando H, Masuda Y, Kitagawa J. Neuron-Glia Crosstalk and Neuropathic Pain: Involvement in the Modulation of Motor Activity in the Orofacial Region. Int J Mol Sci. 2017; 18(10). 10.3390/ijms18102051 PMC5666733 28954391

[22] Watase T, Shimizu K, Komiya H, Ohara K, Iwata K, Ogiso B. Involvement of transient receptor potential vanilloid 1 channel expression in orofacial cutaneous hypersensitivity following tooth pulp inflammation. J Oral Sci. 2018; 60(1): 8–13. 10.2334/josnusd.16-0854 29479030

[23] Chiang CY, Dostrovsky JO, Iwata K, Sessle BJ. Role of glia in orofacial pain. Neuroscientist. 2011; 17: 303–20. 10.1177/1073858410386801 21512131

[24] Komiya H, Shimizu K, Noma N, Tsuboi Y, Honda K, Kanno K, Ohara K, Shinoda M, Ogiso B, Iwata K. Role of Neuron-Glial Interaction Mediated by IL-1β in Ectopic Tooth Pain. J Dent Res. 2018; 97(4): 467–475. 10.1177/0022034517741253 29131694

[25] Zhang P, Bi RY, Gan YH. Glial interleukin-1β upregulates neuronal sodium channel 1.7 in trigeminal ganglion contributing to temporomandibular joint inflammatory hypernociception in rats. J Neuroinflammation. 2018; 15(1): 117 10.1186/s12974-018-1154-0 29678208 PMC5910598

[26] Simons LE, Moulton EA, Linnman C, Carpino E, Becerra L, Borsook D. The human amygdala and pain: evidence from neuroimaging. Hum Brain Mapp. 2014; 35(2): 527–38. 10.1002/hbm.22199 23097300 PMC3920543

[27] Neugebauer V. Amygdala pain mechanisms. Handb Exp Pharmacol. 2015; 227: 261–84. 10.1007/978-3-662-46450-2_13 25846623 PMC4701385

[28] Neugebauer V, Li W, Bird GC, Han JS. The amygdala and persistent pain. Neuroscientist 2004; 10(3): 221–234. 10.1177/1073858403261077 15155061

[29] Thompson JM, Neugebauer V. Amygdala Plasticity and Pain. Pain Res Manag. 2017; 2017: 8296501 10.1155/2017/8296501 29302197 PMC5742506

[30] Sagalajev B, Wei H, Chen Z, Albayrak I, Koivisto A, Pertovaara A. Oxidative Stress in the Amygdala Contributes to Neuropathic Pain. Neuroscience. 2017; pii: S0306-4522(17)30881-3. 10.1016/j.neuroscience.2017.12.009 29274353

[31] Kiritoshi T, Neugebauer V. Pathway-Specific Alterations of Cortico-Amygdala Transmission in an Arthritis Pain Model. ACS Chem Neurosci. 2018 4 13. 10.1021/acschemneuro.8b00022 PMC6146017 29630339

[32] Avivi-Arber L, Seltzer Z, Friedel M, Lerch JP, Moayedi M, Davis K, Sessle B. Widespread Volumetric Brain Changes following Tooth Loss in Female Mice. Front Neuroanat. 2017; 10: 121 10.3389/fnana.2016.00121 28119577 PMC5220047

[33] Zhang Y1, Mao Z1, Pan L1, Ling Z1, Liu X2, Zhang J1, Yu X1. Dysregulation of Pain- and Emotion-Related Networks in Trigeminal Neuralgia. Front Hum Neurosci. 2018; 12: 107 10.3389/fnhum.2018.00107 29662445 PMC5890150

[34] Milligan ED, Watkins LR. Pathological and protective roles of glia in chronic pain. Nat Rev Neurosci. 2009;10(1): 23–36. 10.1038/nrn2533 19096368 PMC2752436

[35] Piao ZG, Cho IH, Park CK, Hong JP, Choi SY, Lee SJ, et al Activation of glia and microglial p38 MAPK in medullary dorsal horn contributes to tactile hypersensitivity following trigeminal sensory nerve injury. Pain. 2006;121(3): 219–31. 10.1016/j.pain.2005.12.023 16495005

[36] Watkins LR, Milligan ED, Maier SF. Glial activation: a driving force for pathological pain. Trends Neurosci. 2001; 24(8): 450–5. 11476884 10.1016/s0166-2236(00)01854-3

[37] Kosarmadar N, Ghasemzadeh Z, Rezayof A. Inhibition of microglia in the basolateral amygdala enhanced morphine-induced antinociception: Possible role of GABAA receptors. Eur J Pharmacol. 2015; 765: 157–63. 10.1016/j.ejphar.2015.08.027 26297974

[38] Kilkenny C, Browne W, Cuthill IC, Emerson M, Altman DG. National Centre for the Replacement, Refinement and Reduction of Animals in Research. Animal research: reporting in vivo experiments—the ARRIVE guidelines. J Cereb Blood Flow Metab. 2011; 31: 991–3. 10.1038/jcbfm.2010.220 21206507 PMC3070981

[39] Liu Q, Gao Z, Zhu X, Wu Z, Li D, He H, et al Changes in nitric oxide synthase isoforms in the trigeminal ganglion of rat following chronic tooth pulp inflammation. Neurosci Lett. 2016; 633: 240–5. 10.1016/j.neulet.2016.09.041 27687716

[40] Stephenson JL, Byers MR. GFAP immunoreactivity in trigeminal ganglion satellite cells after tooth injury in rats. Exp Neurol. 1995; 131: 11–22. 7895805 10.1016/0014-4886(95)90003-9

[41] Costa KM, Maciel IS, Kist LW, Campos MM, Bogo MR. Pharmacological inhibition of CXCR2 chemokine receptors modulates paraquat-induced intoxication in rats. PLoS One. 2014; 9: e105740 10.1371/journal.pone.0105740 25153082 PMC4143277

[42] Ajima H, Kawano Y, Takagi R, Aita M, Gomi H, Byers MR, et al The exact expression of glial fibrillary acidic protein (GFAP) in trigeminal ganglion and dental pulp. Arch Histol Cytol. 2001; 64: 503–11. 11838710 10.1679/aohc.64.503

[43] Gunjigake KK, Goto T, Nakao K, Kobayashi S & Yamaguchi K. Activation of satellite glial cells in rat trigeminal ganglion after upper molar extraction. Acta Histochem Cytochem. 2009; 42: 143–9. 10.1267/ahc.09017 19918323 PMC2775105

[44] Freitas RD, Costa KM, Nicoletti NF, Kist LW, Bogo MR, Campos MM. Omega-3 fatty acids are able to modulate the painful symptoms associated to cyclophosphamide-induced-hemorrhagic cystitis in mice. J Nutr Biochem. 2016; 27: 219–32. 10.1016/j.jnutbio.2015.09.007 26482705

[45] Kramer PR, He J, Puri J, Bellinger LL. A non-invasive model for measuring nociception after tooth pulp exposure. J Dent Res. 2012; 91: 883–7. 10.1177/0022034512454297 22797321 PMC3420391

[46] Shang L, Xu TL, Li F, Su J, Li WG. Temporal dynamics of anxiety phenotypes in a dental pulp injury model. Mol Pain. 2015; 11: 40 10.1186/s12990-015-0040-3 26122003 PMC4487070

[47] Flake NM, Zweifel LS. Behavioral effects of pulp exposure in mice lacking cannabinoid receptor 2. J Endod. 2012; 38: 86–90. 10.1016/j.joen.2011.09.015 22152627

[48] Gibbs JL, Urban R, Basbaum AI. Paradoxical surrogate markers of dental injury-induced pain in the mouse. Pain. 2013; 154: 1358–67. 10.1016/j.pain.2013.04.018 23719574 PMC3743260

[49] Li JG, Lin JJ, Wang ZL, Cai WK, Wang PN, Jia Q, et al Melatonin attenuates inflammation of acute pulpitis subjected to dental pulp injury. Am J Transl Res. 2015; 7: 66–78. 25755829 PMC4346524

[50] Lin JJ, Du Y, Cai WK, Kuang R, Chang T, Zhang Z, et al Toll-like receptor 4 signaling in neurons of trigeminal ganglion contributes to nociception induced by acute pulpitis in rats. Sci Rep. 2015; 5: 125–49. 10.1038/srep12549 PMC4519790 26224622

[51] Chudler EH, Byers MR. Behavioural responses following tooth injury in rats. Arch Oral Biol. 2005; 50: 333–340. 10.1016/j.archoralbio.2004.08.011 15740712

[52] Goto T, Oh SB, Takeda M, Shinoda M, Sato T, Gunjikake KK et al Recent advances in basic research on the trigeminal ganglion. J Physiol Sci. 2016; 66: 381–386. 10.1007/s12576-016-0448-1 27023716 PMC10717556

[53] Liu H, Zhao L, Gu W, Liu Q, Gao Z, Zhu X, et al Temporomandibular joint inflammation activates glial and immune cells in both the trigeminal ganglia and in the spinal trigeminal nucleus. Mol Pain. 2010; 6: 89 10.1186/1744-8069-6-89 21143950 PMC3017032

[54] Liu H, Zhao L, Gu W, Liu Q, Gao Z, Zhu X, et al Activation of satellite glial cells in trigeminal ganglion following dental injury and inflammation. J Mol Histol. 2018; 49(3): 257–263. 10.1007/s10735-018-9765-4 29516260

[55] Burke NN, Geoghegan E, Kerr DM, Moriarty O, Finn DP & Roche M. Altered neuropathic pain behaviour in a rat model of depression is associated with changes in inflammatory gene expression in the amygdala. Genes Brain Behav. 2013; 12: 705–13. 10.1111/gbb.12080 23957449

[56] Lin CS, Niddam DM, Hsu ML. Meta-analysis on brain representation of experimental dental pain. J Dent Res. 2014; 93: 126–33. 10.1177/0022034513512654 24221915

