# Supplementary material for: Activation of trigeminal ganglion satellite glial cells in CFA-induced tooth pulp pain in rats
Source: PLoS One. 2018 Nov 12;13(11):e0207411. doi: 10.1371/journal.pone.0207411 (PMC6231674; doi:10.1371/journal.pone.0207411)
Supplement: S1 Table — (DOC) [file pone.0207411.s001.doc]

**Supplementary Table 1.** Serum levels of pro-inflammatory cytokines in the different experimental groups.

| **Cytokine** | **Groups and Days after Induction of Tooth Pulp Inflammation** | | | | | | | | | | | | | | | |
| --- | --- | --- | --- | --- | --- | --- | --- | --- | --- | --- | --- | --- | --- | --- | --- | --- |
|  | Naïve | | | | Open | | | | Closed | | | | CFA | | | |
|  | 1 | 2 | 3 | 8 | 1 | 2 | 3 | 8 | 1 | 2 | 3 | 8 | 1 | 2 | 3 | 8 |
| **IL-1β** | ND | | | | ND | | | | ND | | | | ND | | | |
| **TNF** | ND | | | | ND | | | | ND | | | | ND | | | |
| **IL-6** | ND | | | | ND | | | | ND | | | | ND | | | |

IL-1β and TNF: limits of detection from 62.5 pg/ml to 4000 pg/ml. IL-6: limits of detection from 125 pg/ml 8000 pg/ml. ND: not detected.
